# Robotic-Assisted versus Conventional Laparoscopic Approach for Rectal Cancer Surgery, First Egyptian Academic Center Experience, RCT

**DOI:** 10.1155/2018/5836562

**Published:** 2018-09-02

**Authors:** Yasser Debakey, Ashraf Zaghloul, Ahmed Farag, Ahmed Mahmoud, Inas Elattar

**Affiliations:** ^1^Assistant Teacher of Surgical Oncology, National Cancer Institute, Cairo University, Egypt; ^2^Head of Robotic Surgery Unit, National Cancer Institute, Cairo University, Egypt; ^3^Head of Colorectal Surgery Unit, Faculty of Medicine, Cairo University, Egypt; ^4^Associated Professor of Surgical Oncology, National Cancer Institute, Cairo University, Egypt; ^5^Professor of Biostatistics and Cancer Epidemiology, National Cancer Institute, Cairo University, Egypt

## Abstract

**Background:**

Undoubtedly, robotic systems have largely penetrated the surgical field. For any new operative approach to become an accepted alternative to conventional methods, it must be proved safe and result in comparable outcomes. The purpose of this study is to compare the short-term operative as well as oncologic outcomes of robotic-assisted and laparoscopic rectal cancer resections.

**Methods:**

This is a prospective randomized clinical trial conducted on patients with rectal cancer undergoing either robotic-assisted or laparoscopic surgery from April 2015 till February 2017. Patients' demographics, operative parameters, and short-term clinical and oncological outcomes were analyzed.

**Results:**

Fifty-seven patients underwent permuted block randomization. Of these patients, 28 were assigned to undergo robotic-assisted rectal surgery and 29 to laparoscopic rectal surgery. After exclusion of 12 patients following randomization, 45 patients were included in the analysis. No significant differences exist between both groups in terms of age, gender, BMI, ASA score, clinical stage, and rate of receiving upfront chemoradiation. Estimated blood loss was evidently lower in the robotic than in the laparoscopic group (median: 200 versus 325 ml, p= 0.050). A significantly more distal margin is achieved in the robotic than in the laparoscopic group (median: 2.8 versus 1.8, p< 0.001). Although the circumferential radial margin (CRM) was complete in 18 patients (85.7%) in the robotic group in contrast to 15 patients (62.5%) in the laparoscopic group, it did not differ statistically (p=0.079). The overall postoperative complication rates were similar between the two groups.

**Conclusion:**

To our knowledge, this is the first prospective randomized trial of robotic rectal surgery in the Middle East and Northern Africa region. Our early experience indicates that robotic rectal surgery is a feasible and safe procedure. It is not inferior to standard laparoscopy in terms of oncologic radicality and surgical complications. Organization number is IORG0003381. IRB number is IRB00004025.

## 1. Background

Over the past two decades, rectal cancer management underwent an immense wave of change from various perspectives. There was a smart shift from open to minimally invasive and robotic techniques, a worldwide application of neoadjuvant multimodal chemoradiation therapy for locally advanced stage disease, as well as optimization of surgical technique with nerve preservation together with the introduction of total mesorectal excision (TME) which were all largely happening in the preceding 10–15 years. The first colorectal laparoscopic procedure was operated upon by Jacobs in 1991 [1]. A decade later, in 2001, the robotic system was introduced to colorectal surgery [2].

To date, laparoscopic surgery has been acknowledged as a safe and effective modality of rectal cancer surgery. However, a randomized controlled multicenter trial has recently suggested that the use of laparoscopic surgery in T3/T4 tumors may result in incomplete resection, affecting the oncological outcome in this group of patients [3]. The challenges of an incomplete TME in laparoscopic surgery are often encountered when faced with anatomical difficulties, for instance, a narrowed male pelvis, bulky tumor, and obese patients. Robotic rectal surgery might be the answer to this quandary.

Why robotics? The company of The da Vinci Surgical System gives many justifications encompassing the high definition 3D image, the wrist-like function, filtration of tremors, microanastomosis, motion scaling, and tele-surgery. Still, we do believe that there are certain perceived advantages during surgery. First is the optimal, stable operative view; the surgeon is no longer assistant-dependent. Second is the high definition 3D image with superior visualization and differentiation between things to be removed from those to be preserved. Moreover, the surgeon's wrist is now incorporated in the technology and he or she would move freely in space deep in the pelvis, a place he could otherwise not get to. Add to that, the countertraction, using the third robotic instrument attached to the fourth robotic arm, with optimal direction and force, may be an operator.

With minimally invasive surgery making headway, we believe that robotic surgery will provide the next major step forward in the treatment of rectal cancer. Like it or not, robotic systems have already revolutionized the surgical field, proving its advantage over laparoscopic techniques in terms of superior visualization, enhanced motion, ergonomics, and comparable clinical outcome.

## 2. Methods

This is a prospective randomized controlled study that was conducted on all patients of both sexes and definite age group attending the National Cancer Institute and with adenocarcinoma of the rectum located within 15 cm from the anal verge who were eligible to be included in the study. Tumor localization was categorized as the upper rectum (distal border of tumor is from 10 to 15 cm from the anal verge), middle rectum (5 to 10 cm from the anal verge), or lower rectum (less than 5 cm from the anal verge) as measured by colonoscopy and digital rectal examination. Patients were classified into two groups: robotic-assisted rectal surgery “the robotic system that we use is the da Vinci Si (Intuitive Surgical, Inc., Sunnyvale, CA)” and conventional laparoscopic rectal surgery. Baseline demographics (gender, age, ASA, BMI), preoperative data (distance of the tumor from the anal verge, clinical stage, whether preoperative chemoradiation “CRT”, presence of residual tumor after CRT), intraoperative data (preparation time, actual operative time, estimated blood loss, and conversion rate to open surgery), postoperative data (pathological stage, number of harvested lymph nodes, macroscopic completeness of resection in the form of proximal margin, distal margin, and circumferential radial margin), and immediate postoperative outcome within one month (days of return of bowel function, days of hospital stay, complications, if any, like anastomotic leakage, ileus, wound problems and others, rate of reoperation, rate of readmission, and 30-day mortality) were analyzed and compared.

The criteria for patients selection were the following: histological diagnosis of adenocarcinoma of rectum, no anesthesiological contraindications to minimally invasive surgery, age ≤ 75 years, ASA ≤ 2, and the procedures performed by the same surgical team. Patients with metastatic disease, malignant bowel obstruction (MBO), and unresectable tumor were excluded from our study.

Preoperative workup (endoscopy with biopsies, radiological imaging including pelvic MRI, liver ultrasound, chest X-ray, and routine abdominal and digital rectal examinations) was routinely carried out. The assignment of patients to either group was done by a permuted block randomization. It was an open-labeled study; i.e., patients, investigators (surgeons, researchers), and data collectors knew which procedure will be done to which patients. The study was approved by the Institutional Review Board (IRB) at NCI, Cairo University. All patients provided written informed consent.

Concerning preoperative preparation; first, mechanical bowel preparation was performed preoperatively with rectal enemas for all patients. A single preoperative dose of antibiotics (oral ciprofloxacin 750 mg and intravenous neomycin 1 gram) was given. Second, from midnight prior to surgery, patients did not receive medications known to cause long-term sedation. Third, for prophylaxis against thromboembolism, subcutaneous enoxaparin 40 mg was given 12 hours before the expected time of the procedure. Fourth, patients received single-dose antibiotic prophylaxis against both anaerobes and aerobes about 1 hour before surgery. Finally, solid diet stopped the day before surgery with no starvation policy as fasting is just for 4 hours for liquids before the procedure.

## 3. Definitions

Anterior or low anterior resection is defined when the anastomosis is performed above or below the peritoneal reflection, respectively. In ultra-low anterior resection the entire rectum is removed and a coloanal anastomosis is performed. Preparation time is calculated from induction of anesthesia to console and the actual operative time from console to skin closure. Stage migration occurs whenever the final pathological stage differs from the preoperative clinical stage. Operative morbidity is marked by complications in the form of wound infection, ileus. Clinical anastomotic leakage was considered to be present, if any of the following features were observed: presence of peritonitis caused by anastomotic dehiscence, presence of feculent substances coming out through the pelvic drain, or the presence of a pelvic abscess with demonstration of anastomotic leak by rectal examination or contrast study.

## 4. Work Description [4]

### 4.1. Operation Room Arrangement

Whichever robotic or laparoscopic is performed, certain principles are adopted: first, patient positioning in a manner precluding pressure or nerve injury so all pressure points were adequately padded; second, patient fixation to the operating table to prevent sliding, so chest strap is placed superior to the xiphoid process and leg strap is also applied over the pneumatic calf; and third, freeing the operative field by positioning IV lines, cardiac monitoring leads, and urinary catheter in a manner that they do not obstruct the surgical team's operative field.

### 4.2. Patient Positioning

**Figure 1:**
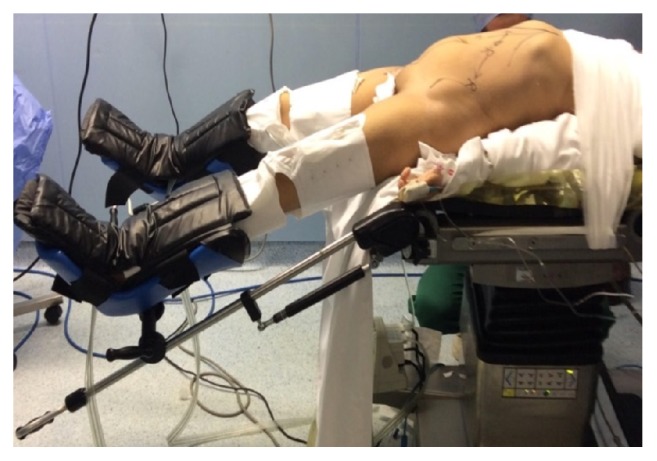
Modified Lloyd-Davis position.

The patient is positioned in a modified lithotomy position with both arms tucked. The patient's abdomen and pelvis are prepared from the xiphoid process to the pubic symphysis and from the right posterior axillary line to the left posterior axillary line. Left docking with an oblique angle (over the hip) is always adopted after the patient is placed in Trendelenburg with the right side tilted downward and the left leg lowered enough to avoid collision during docking,***Figure 1***.

### 4.3. Mark-Up and Trocar Placement

**Figure 2:**
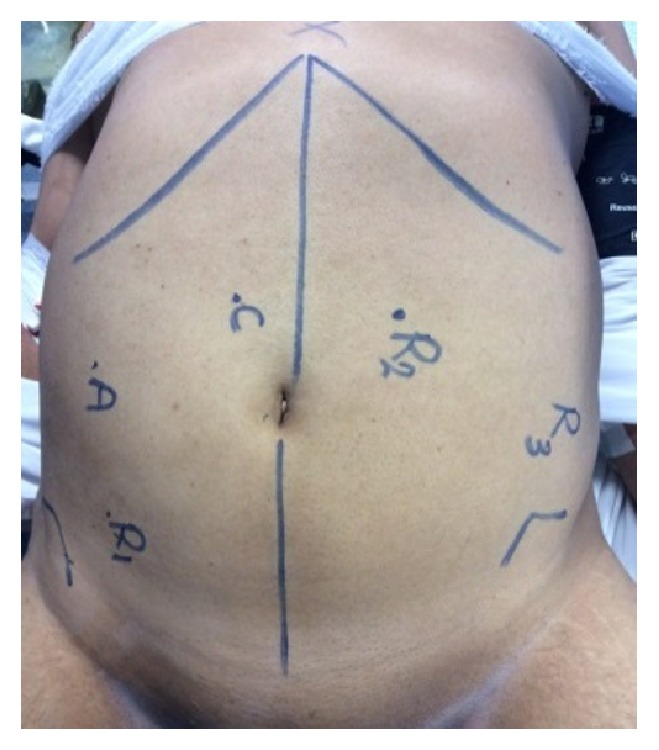
Mark-up.

We adopted a totally robotic approach, so trocars were placed in the right and left lower quadrants (arms 1 and 2, respectively), approximately at the midclavicular lines. Arm 3 is placed in the left lateral abdomen, just superior to anterior iliac spine. An additional 5-mm trocar (assistant port) is placed in right upper quadrant,***Figure 2***. We avoid placing the trocars too far laterally (particularly in a male patient) because collisions with the lateral pelvic sidewall will make the low pelvic dissection difficult. In the midline just superior and to the right of the umbilicus, we place the camera port which is always a 30-degree camera. Additional trocars are placed once insufflation is achieved to ensure the appropriate 8–10 cm distances between ports.

### 4.4. Robotic Technique

#### 4.4.1. Abdominal Phase (Part 1)

The patient is placed in steep Trendelenburg position with the right side tilted downwards allowing the small bowel and the greater omentum to be reflected toward the right upper quadrant and the liver. We prefer in female patients to put a suture transabdominally straight forwardly through the fundus of the uterus to suspend the uterus in order to get an unhampered see into the pelvis. A monopolar scissors is at first utilized in arm 1, fenestrated bipolar forceps are utilized in arm 2, and a double-fenestrated atraumatic bowel grasper is set in arm 3. Utilizing arm 3, the rectosigmoid junction is gotten a handle on. Instead, a right hand may get a handle on this zone, permitting arm 3 to get a handle on more distally on the rectum to give us extra traction.

Dissection is conveyed upwards along the posterior part of the mesorectum toward the IMA, taking consideration not to break the fascia propria of the rectum and avoiding damage to the superior hypogastric nerves. We dissect specifically underneath the inferior mesenteric artery course and above the retroperitoneal fascial planes, the left ureter and gonadal vessels. The ureter and the gonadal vessels could be distinguished simply over the lateral pelvic wall and the pelvic brim. The IMA is elevated from the retroperitoneum and the ureter and gonadal vessels can be effortlessly swept bluntly posteriorly to keep up their situation inside the retroperitoneum, back and lateral to the IMA. We either control the IMA, IMV with clips, sutures, or sometimes Harmonic. The colonic mesentery would now be able to be lifted off the retroperitoneum proximally and along the side. The extent of dissection is superior to the inferior border of the pancreas and laterally overlying the Gerota's fascia. Now, one can rapidly disengage the lateral peritoneal connections to the left colon and sigmoid by beginning at the pelvic brim and continued proximally toward the splenic flexure.

#### 4.4.2. Abdominal Phase (Part 2)

In part 2 of the abdominal phase we deal with freeing the left colonic flexure. A double-fenestrated atraumatic grasper is placed in arm 1; a monopolar curved scissors and a bipolar fenestrated grasper are placed in arms 2 and 3 as required to help mobilizing the splenic flexure in a medial-to-lateral approach keeping the patient in the same position.

#### 4.4.3. Pelvic Phase

We commonly start this dissection along the posterior, right side and proceed caudally, arm 2 pushes the rectum toward the patient's left side, and arm 1 is utilized to perform the dissection which is continued as far to the left pelvic side wall as conceivable to diminish what is needed to be dissected from the patient's left side. The posterior dissection is continued distally just behind the fascia propria of the rectum leaving behind the fascia of the neurovascular corridor intact to protect the hypogastric nerve plexuses. Upon approaching the lateral stalks, they are cut with monopolar cautery. Attention is then turned towards the anterior dissection. The assistant withdraw the rectum down and outside the pelvis. Arm 3 aids tension anterior at the level of the seminal vesicle or posterior vagina by retracting it superiorly and anteriorly. In patients with a tumor involving the posterior rectal wall, our dissection plane is always behind the Denonvilliers' fascia to get it out with the specimen. With anterior-based tumors, our dissection plane is almost always anterior to it.

#### 4.4.4. Anastomotic Technique

If the ECHELON FLEX™ 45 mm stapler can be applied with adequate margin, a double-stapled anastomosis is performed, the rectum was transected with the endoscopic stapler and the specimen was retrieved through a Pfannenstiel or a small incision in the left iliac fossa in most cases. An intracorporeal anastomosis is performed with transanal insertion of a circular stapler. The anastomosis is routinely checked by transanal insufflation of air. When the endoscopic stapler could not be applied with an adequate margin below the tumor, transanal resection and coloanal hand-sewn anastomosis is performed

A diverting loop ileostomy is routinely used in patients with low (below the anterior peritoneal reflection) anastomoses or those patients who received preoperative radiation therapy. Consideration for ileostomy reversal occurs 3 months after the index operation or once any potential adjuvant therapy is completed.

#### 4.4.5. Postoperative Policy

We adopted during this work an enhanced recovery care protocol for patients following either robotic or laparoscopic approach for rectal cancer. Preoperative counseling, adequate fluid and pain management, early feeding, and mobilization following surgery were implemented. The patients were discharged when they fulfilled the discharge criteria which include tolerance to oral diet, adequate pain control with oral analgesics, patient ambulating independently, afebrile patient without tachycardia, nonrising leucocyte count or C-reactive protein (CRP), and adequate home support with ability to take care of the stoma.

## 5. Statistical Analysis

Data management and analysis were performed using Statistical Package for Social Sciences (SPSS) vs. 23. Numerical data were summarized using medians and ranges. Categorical data were summarized as numbers and percentages. Numerical data were explored for normality using Kolmogorov-Smirnov test and Shapiro-Wilk test. Exploration of data revealed that the collected values were not normally distributed. Comparisons between two groups were done by Mann–Whitney test. Chi-square or Fisher's tests were used to compare between the groups with respect to categorical data, as appropriate. All p-values are two-sided. P-values < 0.05 were considered significant.

## 6. Results

**Figure 3:**
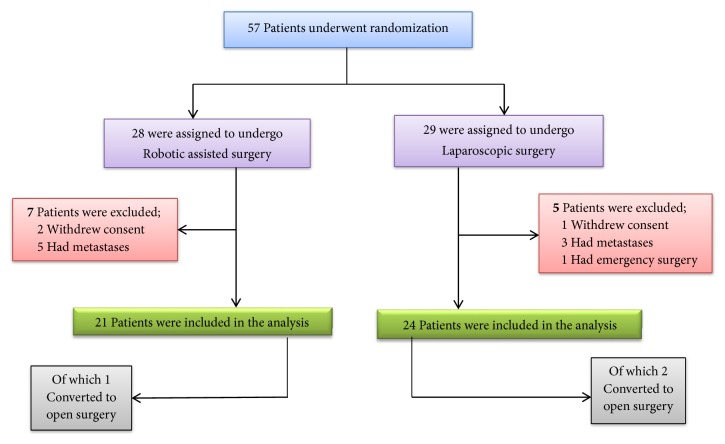
Patient enrollment.

From April 2015 till February 2017, a total of fifty-seven patients with rectal cancer underwent permuted block randomization,***Figure 3***. Of these patients, twenty-eight were assigned to undergo robotic-assisted rectal surgery and twenty-nine to undergo laparoscopic rectal surgery. After the exclusion of twelve patients following randomization for different reasons such as consent withdrawal, emergency surgery, or presence of metastasis, forty five patients (twenty-one in the robotic surgery group and twenty-four in the laparoscopic surgery group) were included in the analysis.

Patient demographic data and characteristics are shown in***Table 1***. There were no significant differences between the groups in terms of age, gender, BMI, ASA score, clinical stage, distance of the tumor from the anal verge, rate of receiving upfront chemoradiation, and rate of no residual lesions after such preoperative therapy. The median age of patients in the robotic surgery group was 53.4 years (range from 32 to 67), while that of those in the conventional laparoscopic surgery group was 50.3 years (range from 36 to 64) (P = 0.241). 47.6% of the patients in the robotic group have BMI below 30 kg/m2, while the other 52.4% have BMI above 30. On the other hand, most of the patients in the laparoscopic group (66%) have BMI above 30, while the remaining 33% were below 30 kg/m2 BMI (P= 0.329). There was a significant difference in terms of sex, albeit more male patients underwent laparoscopic surgery (42.4% in the robotic group versus 54.2% in the laparoscopic group).

Twelve patients in the robotic group received upfront therapy (57.1%) compared to eleven patients (45.8%) in the laparoscopic group with p value 0.449 and only one patient in each group showed complete clinical and radiological response.

Operative data are compared and recorded in Table 2. In the robotic-assisted surgery group, nine patients underwent anterior resection (42.9%), seven underwent low anterior resection (33.3%), four underwent ultra-low anterior resection (19%), and only one patient (4.8%) underwent abdominoperineal resection (APR). On the other side, the laparoscopic group, thirteen patients underwent anterior resection (54.2%), seven underwent low anterior resection (29.1%), one underwent ultra-low resection (4.2%), and finally four underwent APR (12.5%). The preparation time (time from induction of anesthesia to console) was significantly longer in the robotic group with a median of fifty-five minutes compared to twenty-eight minutes in the laparoscopic group with a p value less than 0.001. Similarity, the actual operative time (time from console to wound closure) was also longer in the robotic group with a median of 201 minutes in contrast to 134.5 minutes in the laparoscopic group with a statistically significant p value of less than 0.001. Although not clinically obvious, the estimated blood loss was statistically significantly lower in the robotic group than in the laparoscopic group with a median of 200 ml versus 325 ml, respectively, with a p value of 0.050. Despite our initial experience in robotic colorectal surgery only one case required conversion to open surgery compared to two cases in the laparoscopic group. Five cases had stage migration in the robotic group between stages I, II, and III in comparison to eight cases in the laparoscopic group and at the end eleven patients representing 52.4% were in stage II and ten patients (47.6%) were in stage III, in the robotic group. On the other hand, seventeen patients (70.8%) and seven patients (29.2%) in the laparoscopic group were in stages II and III, respectively. The median distal margin was 2.8 cm in the robotic group, while in the laparoscopic group it was 1.8 cm. This was statistically significant with a p value less than 0.001. Although the circumferential radial margin (CRM) was complete in 18 patients (85.7%) in the robotic group in contrast to 15 patients (62.5%) in the laparoscopic group, it did not differ statistically with a p value of 0.079. There was no statistically significant difference in the number of retrieved lymph nodes with a median of fourteen versus thirteen nodes in the robotic and laparoscopic groups, respectively, and a p value of 0.498. In a similar manner, there was no difference between the two groups in the length of the proximal margin.

Postoperatively,***Table 3***, the median time to passage of first flatus was 2 days in the robotic group and 1.6 days in the laparoscopic group; this was significant statistically with a p value of 0.017. The median length of hospital stay (LOS) was 3 days for patients in the robotic group and only 2 days for patients in the laparoscopic group. This was owing to adoption of a fast track protocol, with no significant statistical difference between the two groups, p value 0.116. The overall postoperative complication rates did not differ between the two groups. Anastomosis leakage occurred once in each group; in the robotic group, the leakage was minimal and was managed successfully conservatively, but unfortunately in the laparoscopic one, the patient was 48-year-old female with mid-rectal cancer who received upfront chemoradiation and known to be diabetic and underwent laparoscopic low anterior resection with covering ileostomy. The leakage was discovered on day 1 with fecal matter coming through the drain with rapid deterioration of the general condition. After adequate resuscitation, a laparoscopic exploration revealed posterior wall disruption with peritoneal soiling. After completion of peritoneal lavage, we thought of two options either performing a Hartman's procedure or refashioning the edges and resuturing the two ends, hand sewing and covering the anastomosis with the already present ileostomy and the latter was opted. We thought that adequate lavage, drainage, and the defunctioning ileostomy were sufficient but things do not always go as you wish. The patient was transferred to the ICU, extubated. The next day the general condition continued to deteriorate with tachycardia over 120/min, tachypnea, and metabolic acidosis with rising acute phase reactants. Her duplex study, echocardiography, and cardiac enzymes detected no abnormality. The patient died on day three. In the robotic surgery group, postoperative ileus occurred in two patients compared to three patients in the laparoscopic group and was managed conservatively in both groups. Wound problems in the form of infection or suture disruption (in APR cases) took place in four patients, divided equally between the two groups. One patient had DVT in the robotic group and one in the laparoscopic group complained of erectile dysfunction.

No reoperation or mortality occurred in the robotic surgery group with only one patient readmitted for disrupting perineal wound following abdominoperineal resection. On the other side, one patient was readmitted on postoperative day 3 because of fever and chest infection, also one patient was reoperated upon and unfortunately died.

According to Clavien-Dindo classification of surgical outcomes, complications occurred in six patients in the robotic group and were primarily of grade one,***Table 4***. One case suffered from deep venous thrombosis and managed with anticoagulants. There was evidence of anastomotic leakage with pelvic collection in another one case of this group dealt with conservatively and with image-guided percutaneous drainage. Comparably, seven cases in the laparoscopic group have complications; five of them in grade one and one case have prolonged paralytic ileus necessitating administration of parenteral nutrition. Grade 5 complications occurred once in this study, Table 4

## 7. Discussion

Come closer! Do not shake! Stay there! Turn right! Left! This is one of the real practical differences between robotic-assisted and laparoscopic surgery wherein the surgeon is still an assistant-dependent.

Of no doubt, rectal surgery underwent an immense wave of change from various perspectives since the beginning of the twenty-first century. There was a smart shift from open to minimally invasive and robotic techniques, a worldwide application of neoadjuvant multimodal chemoradiation therapy for locally advanced stage disease, as well as optimization of surgical technique with nerve preservation together with the introduction of total mesorectal excision (TME) which was all largely happening in the preceding 10–15 years.

However, laparoscopic rectal cancer resection is a complex procedure with a long learning curve, and conventional laparoscopic surgery in the pelvis is difficult, particularly in cases of a bulky tumor in the deep male pelvis. Thus, the conversion rate to the open approach in the CLASICC trial was over 30% [5] and in the COLOR II trial it was 17% [6].

For any new operative technique to become an accepted alternative to conventional methods, it must be proved safe and must result in comparable outcomes. For instance, studies have emerged since the adoption of laparoscopy for colorectal operations that have shown that it can shorten hospital stay, yielding oncologically adequate resection, with no evident differences in postoperative complications or hospital mortality when compared with a traditional open approach. Because of studies like these, laparoscopy is now considered a reasonable alternative to an open approach in colorectal resection [7, 8]. Moreover, other reviews have already demonstrated robotic rectal surgery to be safe and feasible [9–11], although there are no published studies demonstrating its superiority over the laparoscopic approach mainly due to the lack of randomized control trials.

In our study, there was no significant difference between the robotic and laparoscopic groups in terms of age, gender, BMI, ASA, distance from the anal verge, preoperative clinical stage, and rate of upfront chemoradiation. The operations in the current study were performed by a group of experienced laparoscopic surgeons and honesty considered beginners robotic surgeons. Not surprisingly, we admitted that laparoscopic proctectomy is a challenging particularly with bulky tumors in males with narrow pelvis and required complicated maneuvers to reach the extremes of the pelvis. The sphincter-preserving rate was high in the robotic-assisted group 19% compared to 4.2% in the laparoscopic group and abdominoperineal resection was performed in only one patient (4.8%) in the robotic-assisted group in contrast to nearly triple percentage (12.5%) in the laparoscopic group. Such difference may be attributed to the technical characteristics of the robot platform, most importantly, the optimal stable operative pelvic view, the endowrist function, and the countertraction with optimal direction and force using the great third robotic instrument attached to the fourth robotic arm; these are certainly advantageous when compared with the conventional rigid laparoscopic instruments when the pelvis is addressed during surgery.

As regards the conversion rate, only one robotic case was converted to open approach due to bulky mid-rectal tumor in a very narrow male pelvis with no access to reach a comfortable distal margin; it is worth mentioning that it was the second robotic case in the study. On the opposite side, two cases were converted from the laparoscopic approach to the open one. Even though the conversion rate was almost doubled in the laparoscopic approach, this could not be evident statistically due to the small number of cases but resembles the results of Ielpo and associates [12] who suggested that the robotic approach has lower conversion rates when the tumor location requests a low anterior resection and as a consequence, the operation is technically more challenging. In converted cases being associated with greater morbidity and tumor recurrence [13], robotic surgery could provide better oncologic long-term results as well as decreased perioperative morbidity.

**Figure 4:**
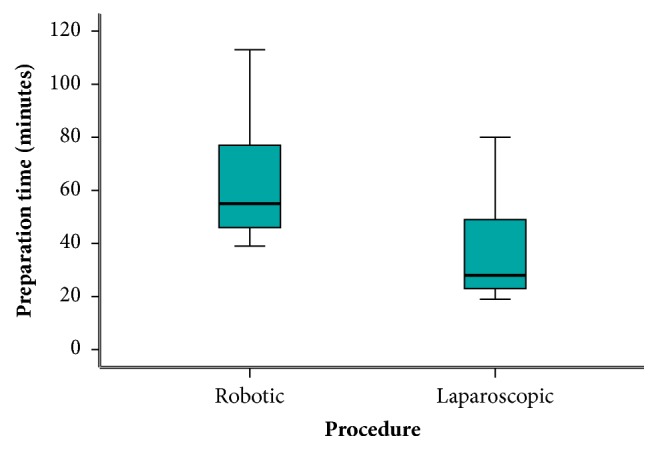
Preparation time.

**Figure 5:**
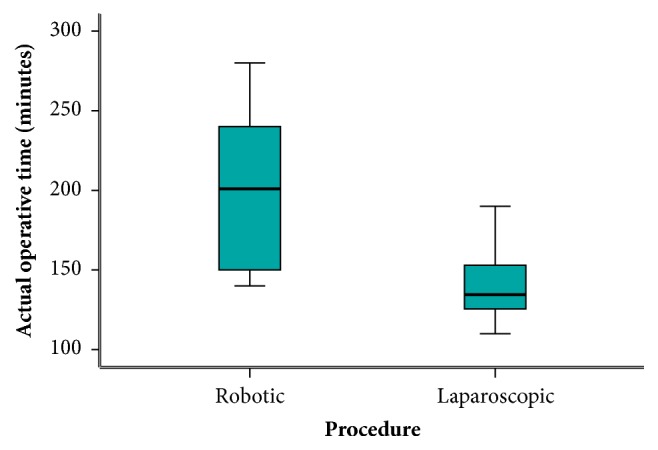
Actual operative time.

An obvious finding of this trial is the longer preparation time*** (Figure 4***) (median time 55 versus 28 minutes) and actual operative time*** (Figure 5***) (201 versus 134.5 minutes) of robotic surgery and laparoscopic surgery, respectively, with a very significant p value in both times of less than 0.001. These results are justified by the time used to dock and undock the robotic system, even in presence of an experienced surgical team. It happens not infrequently that patients on the surgical table require different positions, so the robotic cart needs to be undocked and redocked several times during the same procedure. This extends the operative time. Undoubtedly, the standardization of step by step surgical procedures together with the continuous training of the whole surgical team (surgeons, nurses, anesthesiologists, and operation room staff) can improve the quality of the operations and lead to a progressive reduction of the operative time. Classically speaking, longer operative time is related to increased morbidity, most likely related to the difficulty of the operation [11]; however, prolonged times in robotic surgery are not associated with increased complications as declared by this study and previously published review and meta-analysis of Luca and associates [14].

This randomized trial reported a significantly lower estimated blood loss after robotic rectal surgery (p value =0.050). A recent case-controlled analysis comparing TME between robotic and laparoscopic methods did not show any significant difference in the amount of blood loss [15]. A separate meta-analysis review reaffirmed these findings [16]. The number of harvested lymph nodes is indispensable in the postoperative tumor staging and hence the right treatment for more patients. Of course, its accuracy increases with the number of nodes retrieved within the surgical specimen and should be at least 12, as settled by the last AJCC [17]. In this work, this target was always respected by the high ligation policy of the IMA, both in the laparoscopic and in the robotic group. The difference between the harvested lymph nodes in the robotic (median: 14, range: 8 to 20) and laparoscopic (median: 13, range 9 to 21) groups was not substantial in our study; p value was 0.498. Comparably, previous studies comparing robotic and laparoscopic rectal cancer surgery have shown no significant differences in the number of lymph nodes harvested [18–22]. Eight cases in our study have less than 12 lymph nodes retrieved in their final surgical specimens of which there are 5 in the robotic group and 3 cases in the other arm. Remarkably, 7 cases have received neoadjuvant chemotherapy and two of which have shown complete radiological response with no residual tumor or induration but only one of these two cases showed complete pathological response.

Tumor response assessment of the other 5 cases after upfront chemoradiation by MRI showed partial regression. Coincidence of good tumor response and the low number of harvested lymph nodes raises the probability of better locoregional disease control rather than poor diligence of the surgical or pathological teams. Unfortunately, the small number of cases stands as an obstacle to draw a definitive commentary.

**Figure 6:**
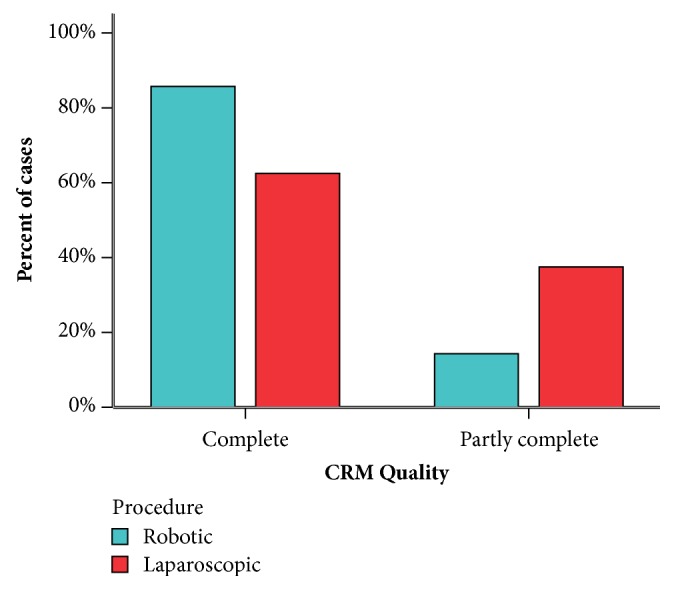
CRM quality.

The quality of the circumferential radial margin together with the distal margin is considered leading parameters in evaluating the treatment of rectal cancer. Findings from the present work seem to determinate the superiority of robotic surgery over the laparoscopic approach,***Figure 6,*** with ability to dissect adequately beyond the lower limit of the tumor in the robotic group (median: 2.8, range: 1.4-4 cm) when compared to the laparoscopic group (median: 1.8, range: 1-2.8). This was evident statistically with a p value of less than 0.001. Even though not statistically significant, achievement of a complete circumferential radial margin, i.e., completeness of the fascia propria of the rectum, occurred in more than 85% of case in the robotic group compared to 62.5% in the laparoscopic group (p value: 0.079). In a similar fashion, a significant wider CRM in their robotic series when compared to the laparoscopic ones was reported by Barnajian [23] and D'Annibale [24].

One of the main benefits of minimally invasive surgery is the early recovery. During this trial we adopted an enhanced recovery protocol wherein sips of water were started on postoperative day (POD) 0, liquid diet on POD 1, and soft diet on POD 2. Patients were nursed in an environment that encouraged independence and mobilization. Patients were strongly encouraged to be out of bed longer than 2 hours beginning on the day after operation. Our discharge criteria included afebrile patient without tachycardia, tolerance of oral feeding, adequate control of pain with oral analgesia, patient ambulating independently, nonrising leucocyte count or CRP, and adequate support at home. Despite being indistinct clinically, there was a significant difference in the day of first flatus in favor of the laparoscopic group 1.6 days compared to 2 days in the robotic group, p value: 0.017.

This study did not show a significant difference between both groups in terms of hospitalization (LOS), complication rate, reoperation, or readmission. Anastomotic leak is the most severe surgical complication in colorectal surgery. Endorsed risk factors for anastomotic leak are cancers located below the peritoneal reflection, irradiated pelvis, obesity, and intraoperative blood transfusions [25, 26]. A covering ileostomy was adopted in presence of any of these hazardous criteria. In this study, the overall anastomotic leakage rates in the robotic and laparoscopic series were similar (4.8% versus 4.2%).

The discern advantage of the robotic high definition 3D image allows better identification of autonomic nerves. Potential sites of nerve damage are the superior hypogastric plexus, leading to ejaculatory dysfunction in males and impaired lubrication in females, and the pelvic splanchnic nerves deep in the lateral pelvic wall leading to erectile dysfunction in men which occurred in one patient in the laparoscopic group representing 4.2% compared to 0% in the robotic group in sight of this study. We adopt a short questionnaire to assess the sexual function including failure of ejaculation or erection and whether failure of the latter is complete or partial i.e., difficulty in penetration or in maintaining erection. We have only one case who complained of partial erectile dysfunction. According to results of the CLASSIC trial [27], autonomic injury risk with sexual dysfunction in males is significantly higher in laparoscopic surgery when compared to the open approach. In the same context, two studies suggested that robotic-assisted rectal surgery is better than conventional laparoscopic surgery in preventing sexual or urinary dysfunction [26, 28].

It is worth mentioning than the most important point of strength in this work is being randomized by permuted block randomization trying to minimize the selection bias.

We have also to mention about the weakness of this study. First, the small sample size prevents us from drawing proper conclusions, especially for each type of procedure. Moreover, this study did not focus properly in the functional outcome aside from a short follow-up period of one month. Finally, the different stand points of experience between robotic and laparoscopic rectal surgery among surgeons may fail to signify the great technical characteristics of the robotic platform.

## 8. Conclusion

To our knowledge, this is the first prospective randomized trial of robotic rectal surgery in the Middle East and Northern Africa region.

Our early experience indicates that robotic rectal surgery is a feasible and safe procedure. It is not inferior to standard laparoscopy in terms of oncologic radicality and surgical complications.

This randomized control trial on 45 patients has demonstrated that the distal margin and the quality of the CRM with the robotic system are better than conventional laparoscopy but with an obvious weak spot of longer operative time. Of course more randomized trials with larger sample size are needed to stabilize the robotic approach in rectal cancer surgery. Furthermore, the current commercial robotic platform is best suited for a single quadrant confined dissection. As such, it has some limitations in rectal surgery where more than one quadrant is involved. On the contrary, the perfect stable operative view, the wrist-like function, and the optimal direction and force created by the third robotic instrument might improve the diffusion of this minimally invasive technology in the treatment of rectal cancer.

Finally, it is just a beginning, as we believe; it is very difficult to imagine a surgeon in the further future working without interface.

## Figures and Tables

**Table 1:** Patients demographic data and characteristics.

	Robotic	Laparoscopic	p value
	No. 21	No. 24	
Age	53.4 (32-67)	50.3 (36-64)	0.241
Gender			0.905
Female	10 (47.6%)	11 (45.8%)	
Male	11 (42.4%)	13 (54.2%)	
MBI (kg/m2)			0.329
(i) < 30	10 (47.6%)	08 (33.3%)	
(ii) >/= 30	11 (52.4%)	16 (66.7%)	
ASA score			
(i) Healthy	18 (85.7%)	18 (75%)	
(ii) Mild systemic disease	03 (14.3%)	06 (25%)	
Distance from anal verge			
(i) Upper rectum	9 (42.9%)	13 (54.2%)	
(ii) Middle rectum	10 (47.6%)	8 (33.3%)	
(iii) Lower rectum	2 (9.5%)	3 (12.%)	
Clinical stage			
(i) I	1	4	
(ii) II	15	17	
(iii) III	5	3	
Preoperative chemoradiation	12 (57.1%)	11 (45.8%)	0.449
No residual lesion	01 (4.8%)	01 (4.1%)	

**Table 2:** Operative data.

	Robotic	Laparoscopic	p value
	No. 21	No. 24	
Type of operation			
(i) Anterior resection	9 (42.9%)	13 (54.2%)	
(ii) Low anterior resection (LAR)	7 (33.3%)	7 (29.1%)	
(iii) Ultra-LAR	4 (19%)	1 (4.2%)	
(iv) APR	1 (4.8%)	3 (12.5%)	
Median preparation time (min)	55 (39-113)	28 (19-80)	<0.001
Median actual operative time (min)	201 (140-280)	134.5 (110-190)	<0.001
Median estimated blood loss (ml)	200 (50-650)	325 (100-800)	0.050
Convention to open surgery	1 (4.8%)	2 (8.3%)	
Pathological stage			0.203
(i) II	11 (52.4%)	17 (70.8%)	
(ii) III	10 (47.6%)	07 (29.2%)	
Median proximal margin (cm)	13 (10-20)	15 (11-23)	0.270
Median distal margin (cm)	2.8 (1.4-4)	1.8 (1-2.8)	<0.001
CRM quality			0.079
(i) Complete	18 (85.7%)	15 (62.5%)	
(ii) Partly complete	03 (14.3%)	09 (37.5%)	
Median LN retrieved (no.)	14 (8-20)	13 (9-21)	0.498

**Table 3:** Immediate Postoperative Outcomes.

	Robotic	Laparoscopic	p value
	No. 21	No. 24	
Flatus (median days)	2 (1-4.3)	1.6 (0.5 -5)	0.017
LOS (median days)	3 (2-14)	2 (2-11)	0.116
Complications			0.965
Anastomotic leakage	1 (4.8%)	1 (4.2%)	
Ileus (median days)	2 (9.5%)	3 (12.5%)	
Wound problems	2 (9.5%)	2 (8.3%)	
Others	1 (DVT)	1( erectile dysfunction)	
Reoperation	0	1 (4.2%)	
Readmission	1 (4.8%)	1 (4.2%)	
Death	0	1 (4.2%)	

**Table 4:** Severity of complications according to Clavien-Dindo classification.

Clavien-Dindo	Robotic	Laparoscopic	P Value
Classification	No. 21	No. 24	
No complications	15(71.4%)	18(75%)	0.787
Grade I	4(19.04%) “2 wound infections + 2 ileus”	5(20.83%) “1 erectile dysfunction+ 2 wound infection+2 ileus”	
Grade II	1(4.8%) “DVT”	1(4.2%) “ileus”	
Grade III	1(4.8%) “leakage”	0	
Grade IV	0	0	
Grade V	0	1	

## Data Availability

The datasets generated during the current study are in the Cancer Registry Center of the National Cancer Institute, Cairo University, Egypt, and available upon request.

## Abbreviations

RCT: Randomized controlled trial
BMI: Body mass index
ASA: American Society of Anesthesiologists
CRM: Circumferential radial margin
TME: Total mesorectal excision
CRT: Chemoradiation therapy
MBO: Malignant bowel obstruction
IRB: Institutional Review Board
NCI: National Cancer Institute
LOS: Length of stay
IMA: Inferior mesenteric artery
CRP: C-reactive protein
SPSS: Statistical package for social sciences
LAR: Low anterior resection
APR: Abdominoperineal resection
DVT: Deep venous thrombosis
POD: Postoperative day.
